# Results from the First 12 Months of the National Surveillance of Healthcare Associated Outbreaks in Germany, 2011/2012

**DOI:** 10.1371/journal.pone.0098100

**Published:** 2014-05-29

**Authors:** Sebastian Haller, Tim Eckmanns, Justus Benzler, Kristin Tolksdorf, Hermann Claus, Andreas Gilsdorf, Muna Abu Sin

**Affiliations:** 1 Department for Infectious Disease Epidemiology, Robert Koch Institute, Berlin, Germany; 2 Postgraduate Training for Applied Epidemiology, Berlin, Germany, affiliated to the European Programme for Intervention Epidemiology Training, European Centre for Disease Prevention and Control, Stockholm, Sweden; Inserm & Universite Pierre et Marie Curie, France

## Abstract

**Background:**

In August 2011, the German Protection against Infection Act was amended, mandating the reporting of healthcare associated infection (HAI) outbreak notifications by all healthcare workers in Germany via local public health authorities and federal states to the Robert Koch Institute (RKI).

**Objective:**

To describe the reported HAI-outbreaks and the surveillance system’s structure and capabilities.

**Methods:**

Information on each outbreak was collected using standard paper forms and notified to RKI. Notifications were screened daily and regularly analysed.

**Results:**

Between November 2011 and November 2012, 1,326 paper forms notified 578 HAI-outbreaks, between 7 and 116 outbreaks per month. The main causative agent was norovirus (n = 414/578; 72%). Among the 108 outbreaks caused by bacteria, the most frequent pathogens were *Clostridium difficile* (25%) *Klebsiella* spp. (19%) and *Staphylococcus* spp. *(*19%). Multidrug-resistant bacteria were responsible for 54/108 (50%) bacterial outbreaks. Hospitals were affected most frequently (485/578; 84%). Hospital outbreaks due to bacteria were mostly reported from intensive care units (ICUs) (45%), followed by internal medicine wards (16%).

**Conclusion:**

The mandatory HAI-outbreak surveillance system describes common outbreaks. Pathogens with a particular high potential to cause large or severe outbreaks may be identified, enabling us to further focus research and preventive measures. Increasing the sensitivity and reliability of the data collection further will facilitate identification of outbreaks able to increase in size and severity, and guide specific control measures to interrupt their propagation.

## Background

Healthcare-associated infections (HAIs) are among the most common complications of hospital stays [1]. It is estimated that HAIs are responsible for 10,000 to 15,000 fatalities per year in Germany [2]. In a recent European point prevalence survey of HAIs the total annual number of patients with an HAI in acute care hospitals in Europe was estimated to be 3.2 million [3].

Studies have shown that surveillance within hospitals may lead to a reduction of the incidence of HAI [4]–[8]. Furthermore, as outbreaks of HAIs are potentially preventable, an early outbreak detection and control may decrease morbidity, mortality and costs [9], [10].

In 2001, the German Protection against Infection Act (*Infektionsschutzgesetz*, IfSG) was enacted, regulating mandatory surveillance in Germany. An electronic surveillance system (SurvNet@RKI) was developed to establish a national surveillance system for notifiable diseases to support the communication of notifications between local, federal and state institutions [11]. HAI-outbreaks were notifiable to the responsible local public health authorities since 2001, but information on HAI-outbreaks were generally not forwarded to the German national public health institute, the Robert Koch Institute (RKI), and there was no common identifier for HAI-outbreaks in SurvNet@RKI.

In August 2011, an amendment to the IfSG was passed mandating communication of all HAI-outbreaks by all healthcare workers in Germany via local public health authorities and federal state authorities, to RKI, regardless of the causative pathogen or disease. As a direct response, we developed a national HAI-outbreak surveillance system to report the incidence, severity and scale of all HAI-outbreaks in Germany in order to identify supra-regional outbreaks and reduce the incidence and severity of HAI-outbreaks.

### Objective

We describe the implementation and structure of HAI-outbreak surveillance in Germany and present the results of the first 12 months of systematic data collection and analysis.

## Methods

### Definition of HAI-outbreaks

According to the IfSG, which regulates mandatory notifications, a HAI-outbreak must be notified when two or more epidemiologically-linked nosocomial infections are identified. Notifications must include information on the number of symptomatic infected and colonised patients and fatalities.

According to IfSG, a nosocomial infection is defined as a local or systemic reaction to a pathogen or its toxin with an epidemiological link to any medical procedure. Colonisation is defined as the presence of microorganisms on skin, mucous-membranes in open wounds, in excretions or secretions, but with no resultant adverse clinical signs or symptoms, as per the CDC/NHSN surveillance definition of HAIs [12]. All data on fatalities occurring in patients belonging to an HAI context of the outbreak are transmitted. All healthcare workers in Germany must notify the local public health authority of outbreaks meeting the HAI-outbreak definition. The local public health authorities must communicate the required information, and consider initiating an outbreak investigation.

We piloted data collection on a standardised paper form prior to implementation of the electronic surveillance system. Development of these paper forms incorporated regular feedback from federal public health departments and local public health authorities. HAI-outbreak surveillance was discussed during weekly telephone conferences and biannual meetings with federal state authorities.

The paper form consists of 2 parts:

1. Aggregated data: number of total cases, number of colonised, symptomatic infected and fatal cases, source, infectious agent, multidrug resistance, transmission and institution.
2. Individual case data: clinical diagnosis, date and microbiological diagnosis.

Only anonymous patient information is transmitted according to IfSG.

Follow-up reports must be sent by the responsible local public health authority to RKI whenever changes occur, e.g. case numbers. A final notification should be communicated indicating the end of the outbreak, also clarifying whether suspected outbreaks could be confirmed. If there were no changes after the initial notification, the final notification was not mandatory. Multidrug resistance was not further defined, and so was dependant on the results of susceptibility testing and classification by those reporting the outbreak.

### Data Entry, Data Transmission and Proceeding

Data was entered using an EpiData (http://www.epidata.dk/) data entry mask. Two investigators screened these data to identify duplications. Notifications (i.e. first, follow-up and termination) belonging to a single outbreak were identified by grouping pathogens and geographical regions. Outbreaks not meeting the case definition were excluded. The overall reported number of affected cases was not always identical to the sum of colonised, symptomatic infected and fatal cases.

Standardised paper forms were sent to RKI by fax or email. Notifications were required to be forwarded to the federal state public health authorities no later than on the 3^rd^ working day of the week following the notification. Federal state authorities then transmitted the notification to RKI within one week. At RKI, notifications were screened daily and federal state authorities were contacted in the case of severe outbreaks (for example, outbreaks characterised by an unusually high number of cases or high case-fatality, or antimicrobial resistance patterns with limited treatment options), or a high risk of further spread, or if important data were missing or implausible. HAI-outbreaks may also be discussed during the weekly telephone conference organised by RKI with the federal state authorities [13].

### Ethics Statement

As a federal law, the IfSG regulates the prevention and management of infectious diseases in humans. In order to guarantee confidentiality, the IfSG requires that data on notified HAI-outbreaks is reported anonymously to the national authority (RKI).

### Supra-regional Outbreaks

Supra-regional outbreaks were defined as outbreaks affecting more than one administrative district in Germany. When supra-regional outbreaks were reported or suspected, we contacted federal state authorities, encouraged analysis of isolates in the respective national reference laboratory(s) and offered further advice and an outbreak investigation team, available upon request by the responsible public health authorities.

### Timeliness

Timeliness was estimated by subtracting the date of notification to the local public health authority from the date the RKI received the report.

The analysed time frame included notifications received at RKI between November 01, 2011 and October 31, 2012.

### Statistical Analysis

Descriptive data analysis was performed using STATA (Version 12; *STATA* Corp., TX, USA).

### Feedback

Surveillance results are made available to stakeholders by the national annual epidemiological report on infectious diseases in Germany (http://www.rki.de/DE/Content/Infekt/Jahrbuch/jahrbuch_node.html [14]).

## Results

**Figure 1:**
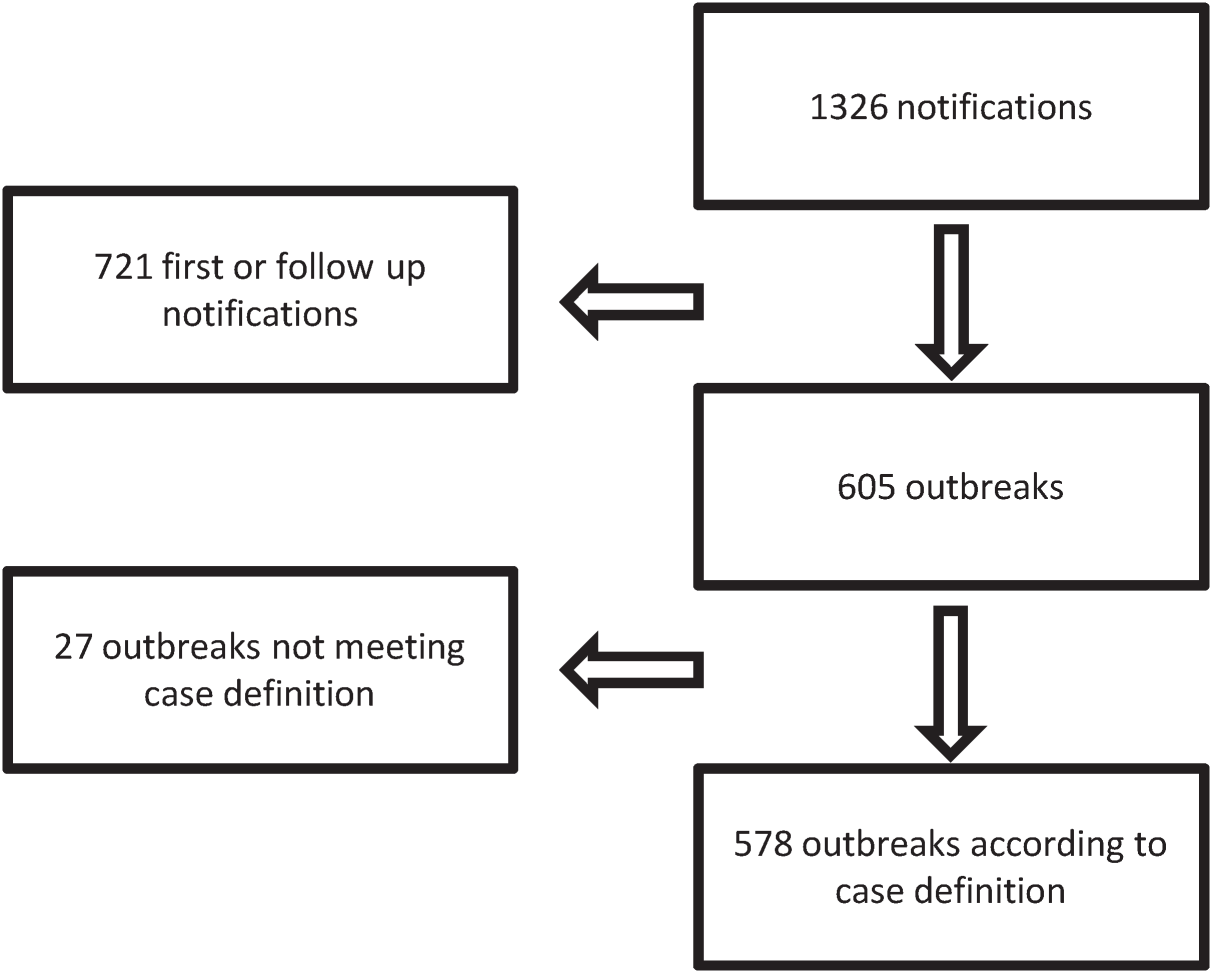
Number of received notifications and their relation to outbreaks matching the case definition – Mandatory outbreak reporting in Germany, 1 November 2011 to 31 October 2012.

During the first 12 months of surveillance we received 1,326 paper forms relating to 605 outbreaks. Up to 12 follow-up notifications per outbreak were communicated per outbreak. Twenty-seven outbreaks had to be excluded because they did not meet the case definition (i.e. less than 2 individuals symptomatic infected or not related to a medical procedure) therefore 578/605 outbreaks met the case definition and remained for further analysis (Figure 1). Among these 578 outbreaks, 74% were due to viral pathogens, 19% were due to bacteria and <1% were due to fungi. In 7% of outbreaks, the pathogen remained unknown (Table 1).

**Table 1:** Pathogens identified in reported HAI-outbreaks (n = 578), Germany, 1 November 2011 to 31 October 2012.

Pathogen		Outbreaks	Cases	Colonisations	SymptomaticInfections	Fatalities
**Virus**	norovirus	414 (96%)	7384 (11.5; 2–229)	0	7380 (11.5; 2–123)	4 (0; 0–2)
	rotavirus	10 (3%)	96 (5; 3–29)	0	95 (4.5; 3–29)	1 (0; 0–1)
	respiratory syncytialvirus	2 (1%)	23 (11.5; 5–18)	0	20 (10; 5–15)	3 (0–3)
	influenza-A-virus	2 (1%)	26 (13; 7–19)	0	26 (13; 7–19)	0 (0)
	adenovirus	2 (1%)	62 (31; 22–40)	0	62 (31; 22–40)	0 (0)
	**virus total**	**430 (100%)**	**7591 (11; 2–229)**	**0**	**7583 (11; 2–229)**	**8 (0; 0–3)**
**Bacteria**	*Clostridium difficile*	27 (25%)	119 (3; 2–15)	0 (0)	105 (3; 1–13)	16 (0; 0–4)
	*Klebsiella* spp.	21 (19%)	212 (5; 2–63)	55 (0; 0–21)	85 (3; 2–15)	19 (0; 0–6)
	*Staphylococcus* spp.	21 (19%)	102 (4; 2–15)	19 (0; 0–10)	77 (3; 1–11)	5 (0; 0–2)
	*Acinetobacter* spp.	10 (9%)	39 (4; 2–6)	7 (0; 0–4)	31 (3; 1–5)	1 (0; 0–1)
	*Enterococcus* spp.	10 (9%)	43 (3; 2–17)	13 (0; 0–10)	29 (2,5; 1–7)	4 (0; 0–2)
	*Escherichia coli*	7 (7%)	19 (2; 2–5)	0	17 (2; 1–5)	2 (0; 0–1)
	*Serratia marcescens*	4 (4%)	34 (4.5; 3–22)	15 (0.5; 0–14)	16 (3; 3–7)	2 (0.5; 0–1)
	*Enterobacter* spp.	3 (3%)	15 (4; 2–9)	0	14 (3; 2–9)	1 (0.5; 0–1)
	*Stenotrophomonas* spp.	3 (3%)	8 (3; 2–3)	0 (0)	8 (3; 2–3)	1 (0; 0–1)
	*Mycoplasma pneumoniae*	1 (1%)	3 (3)	0 (0)	3 (3)	0 (0)
	*Pseudomonas aeruginosa*	1 (1%)	15 (15)	0 (0)	15 (15)	0 (0)
	**bacteria total**	**108 (100%)**	**609 (4; 2–63)**	**109 (0; 0–21)**	**400 (3; 0–15)**	**51 (0; 0–6)**
**Fungi**	*Aspergillus* spp.	2 (100%)	19 (8–11)	6	13 (2–11)	0 (0)
	**fungi total**	**2 (100%)**	**19 (8–11)**	**6**	**13 (2–11)**	**0 (0)**
**Pathogen unknown**	**total**	**38 (100%)**	**514 (2–52)**	**0**	**507 (1–52)**	**8 (0–6)**

Median and range as min. and max. within outbreaks. Note: Numbers of colonised, symptomatic infected and fatalities may not add up to number of all cases (see discussion). Percentages may not add up to total 100% due to rounding.

**Figure 2:**
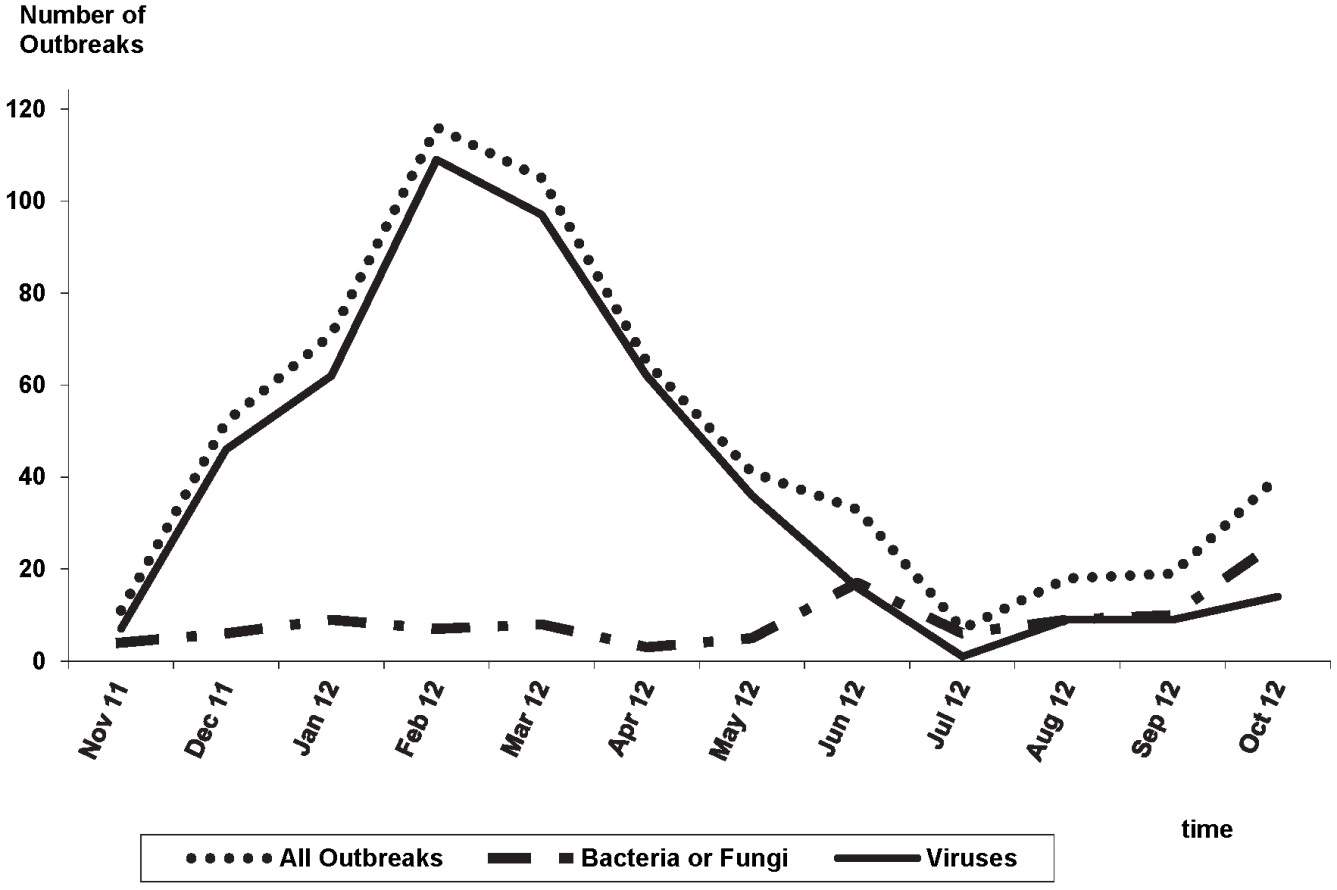
Monthly outbreaks – Mandatory outbreak reporting in Germany, 1 November 2011 to 31 October 2012.

The number of outbreaks notified per month ranged from 7 to 116 outbreaks with a peak in February (Figure 2).

In total, there were 578 outbreaks with 8,733 cases (2–229 patients per outbreak, median: 9) including 67 fatalities (≤6 per outbreak). Bacteria were identified as the causative pathogen in 108/578 outbreaks (19%) with 609 cases (2–63 patients per outbreak, median: 4), including 51 fatalities. Among the 108 outbreaks caused by bacteria, frequent pathogens were: *Clostridium (C.) difficile* (25%) *Klebsiella* spp. (19%) and *Staphylococcus* spp. *(*19%) (Table 1).

No pathogens were identified in 38 mostly gastro-intestinal outbreaks. These events accumulated 514 cases of which 8 were fatal.

Multidrug-resistant organisms (MDROs) were identified as the causative agent for 50% of all outbreaks caused by bacteria (n = 54 of 108) (Table 2). Among the MDROs, the most frequently reported pathogens were Extended-spectrum beta-lactamase (ESBL) *Klebsiella (K.) pneumoniae (n = 6)*, Carbapenem-resistant *K. pneumoniae (n = 9)*, Oxacillin-resistant *Staphylococcus (S) aureus (n = 19)* and ESBL *Escherichia (E.) coli (n = 7)*. Vancomycin resistant Enterococci (VRE) were found in two outbreaks. The antimicrobial sensitivity profile was not specified for 11 outbreaks reported as due to MDROs.

**Table 2:** Number of outbreaks, number of all cases, colonisations, symptomatic infections and fatalities in HAI-outbreaks due to multidrug resistant organisms, Germany, 1 November 2011 to 31 October 2012.

Multidrugresistant organisms	Outbreaks n (%)	Cases n (median;range)	Colonisations n(median; range)	SymptomaticInfectionsn (median; range)	Fatalitiesn (median;range)
*Clostridium difficile*	1 (2%)	3 (3)	0 (0)	3 (3)	0 (0)
*Klebsiella* spp.	15 (30%)	168 (5; 2–63)	43 (0; 0–21)	53 (3; 2–9)	18 (0; 0–6)
*Staphylococcus aureus* (MRSA)	19 (35%)	85 (4; 2–11)	9 (0; 0–7)	73 (3; 1–11)	3 (0; 0–1)
*Acinetobacter* spp.	4 (7%)	16 (4; 3–5)	3 (0; 0–3)	12 (3.5; 1–4)	1 (0; 0–1)
*Escherichia coli*	7 (13%)	19 (2; 2–5)	0 (0)	17 (2; 1–5)	2 (0; 0–1)
*Enterococcus* spp.	5 (9%)	30 (3; 2–17)	13 (3; 0–10)	16 (3; 1–7)	4 (1; 0–2)
*Serratia marcescens*	2 (4%)	26 (13; 4–22)	14 (7; 0–14)	10 (5; 3–7)	1 (0.5; 0–1)
*Enterobacter* spp.	1 (2%)	2 (2)	0 (0)	2 (2)	0 (0)
**Total**	**54 (100%)**	**349 (4; 2–63)**	**82 (0; 0–21)**	**186 (6; 1–11)**	**29 (0; 0–6)**

Note: Numbers of colonised, symptomatic infected and fatalities may not add up to number of all cases (see discussion). Percentages may not add up to 100% due to rounding. MRSA: Methicillin-resistant *Staphylococcus aureus.*

Almost all HAI-outbreaks (97%) were notified by the inpatient care setting, whereas only 1% of outbreaks were notified by the outpatient sector (Table 3). HAI-outbreaks were mainly reported by acute care hospitals (84% of notified outbreaks, Tables 3 and 4).

**Table 3:** Number of outbreaks by healthcare setting and reporting healthcare facility, Germany, 1 November 2011 to 31 October 2012.

Setting		Viruses n(%)	Bacteria n(%)	MDROs n(%)	Fungi n(%)	All outbreaksn (%)
**inpatient/outpatient care**	outpatient care	3 (1%)	0	0 (0%)	0	**3 (1%)**
	inpatient care	451 (96%)	108 (100%)	54 (100%)	2 (100%)	**561 (97%)**
	unknown	14 (3%)	0	0 (0%)	0	**14 (2%)**
	**total**	**468 (100%)**	**108 (100%)**	**54 (100%)**	**2 (100%)**	**578 (100%)**
**healthcare facility**	acute care hospital	382 (82%)	101 (94%)	51 (94%)	2 (100%)	**485 (84%)**
	rehabilitation clinic	23 (5%)	3 (3%)	2 (4%)	0	**26 (5%)**
	long term care facility	47 (10%)	3 (3%)	1 (2%)	0	**50 (9%)**
	medical practice	3 (1%)	0	0 (0%)	0	**3 (1%)**
	unknown/others	13 (3%)	1 (1%)	0 (0%)	0	**14 (2%)**
	**total**	468 (100%)	108 (100%)	54 (100%)	2 (100%)	**578 (100%)**

Note: Percentages may not add up to 100% due to rounding. Multidrug resistant organisms (MDROs).

**Table 4:** Number of outbreaks by reporting hospital ward, Germany; 1 November 2011 to 31 October 2012.

hospital ward(inpatient)	Virusesn (%)	Bacterian (%)	MDROsn (%)	Fungin (%)	All outbreaksn (%)
internal medicine ward	174 (46%)	16 (16%)	2 (4%)	0	**190 (39.2%)**
surgical ward	23 (6%)	12 (12%)	9 (18%)	0	**35 (7.2%)**
hemato-oncology ward	6 (2%)	1 (1%)	0	0	**7 (1.4%)**
intensive care unit	4 (1%)	45 (45%)	30 (59%)	2 (100%)	**51 (10.5%)**
neonatal intensive care unit	4 (1%)	9 (9%)	2 (4%)	0	**13 (2.7%)**
psychiatric ward	32 (8%)	0	0	0	**32 (6.6%)**
geriatric ward	46 (12%)	3 (3%)	1 (2%)	0	**49 (10.1%)**
pediatric ward	6 (2%)	1 (1%)	0	0	**7 (1.4%)**
more than one ward	25 (7%)	2 (2%)	2 (4%)	0	**27 (5.6%)**
others	62 (16%)	12 (12%)	5 (10%)	0	**74 (15.3%)**
**Total**	**382 (100%)**	**101 (100%)**	**51 (100%)**	**2 (100%)**	**485 (100%)**

Note: Percentages may not add up to 100% due to rounding. Multidrug resistant organisms (MDROs).

Ninety-eight outbreaks (17%) provided information about a probable source and/or the route of transmission. Person-to-person transmission was suspected for 82 of these; some were further specified with details such as “having used the same bathroom” (norovirus), or “lack of hand-hygiene amongst healthcare workers and/or patients”.

Environmental investigation identified the causative pathogen in nine outbreaks but it was not further specified where. One outbreak of *Pseudomonas* spp. infections was caused by contaminated water used for mouth rinse; a VRE-outbreak was associated with gastroscopy and colonoscopy; an adenovirus outbreak resulted from contaminated eye drops; an airborne *Aspergillus* outbreak on an intensive care unit (ICU) followed construction work and a foodborne norovirus outbreak occured at a rehabilitation center.

One supra-regional outbreak was detected. It was a large *K. pneumoniae* outbreak with cases in two different hospitals in two different districts. The epidemiological link was the transfer of colonised patients between hospitals.

During the study period, RKI was involved in five HAI-outbreak investigations and provided advice via telephone on several other occasions.

Median time to RKI-notification was 2 days (range 0–136 days) from the time when local public health authorities were notified. Overall data completeness ranged from 70% (“date of first diagnosis”) to 100% (“date of data transmission”).

## Discussion

We describe the systematic collection of HAI-outbreak data in Germany during nationwide, mandatory HAI-outbreak surveillance. The notification of 578 outbreaks within its first year demonstrates that this newly established surveillance was well accepted. Norovirus was the most commonly reported pathogen (n = 414 outbreaks), followed by *C. difficile* (n = 27), *Klebsiella* spp. and *Staphylococcus* spp. (each n = 21), rotavirus, *Acinetobacter* and *Enterococcus* (each n = 10). The surveillance system proved capable of detecting supra-regional outbreaks: it detected one, and subsequent investigations identified the likely transmission route.

Regional differences in outbreak incidence were identified that were likely due to reporting bias rather than true differences in incidence. These were discussed with regional stakeholders during the regular teleconferences, and differences in the practical application of the surveillance protocols were a plausible explanation in many cases (data not shown). We plan to overcome this through further development and distribution of the protocol, and through provision feedback on data quality to regional stakeholders. Hopefully, through standardizing the HAI-outbreak surveillance we encouraged stakeholders to follow recommendations for investigations of HAI-outbreaks in Germany [15], [16].

Reporting of HAI-outbreaks to local public health authorities has been mandatory since 2001, and so we do not expect that significant extra costs were invoked through the establishment of this national surveillance system. An economic investigation of the system’s impact would be a useful component of a full formal evaluation in future.

In a systematic review of outbreak investigations published between 1966 and 2002, Gastmeier et al. reported that *S. aureus* (14.8%) caused most published outbreaks, followed by *Pseudomonas aeruginosa* (8.9%), *K. pneumoniae* (7.1%), and *Serratia marcescens* (6.6%) [17].. Published reports have a tendency for reporting exceptional events, pathogens, and dimensions of outbreaks, whereas mandatory surveillance is more likely to display “reality” [19]. Rhinehart et al. found norovirus (18.2%) to be the most frequent cause of HAI-outbreaks followed by *S. aureus* (17.5%), *Acinetobacter* (13.7%), and *C. difficile* (10.3%) in a survey among US infection preventionists for the years 2008 and 2009 [18]. The relative frequency of the pathogens responsible for HAI-outbreaks may change over time and differ between countries. The relative proportions of outbreak-causing pathogens identified by Rhinehart et al. are similar to our findings. Differences may be explained partly by the emergence of norovirus and *C. difficile* as pathogens causing HAI-outbreaks [20], [21]. Furthermore Rhinehart et al only analysed data from acute care hospitals, whereas the national mandatory HAI surveillance system in Germany includes all healthcare settings, including long-term care facilities. In addition, the high proportion of norovirus outbreaks may partly be explained by norovirus surveillance in Germany, as infections have been mandatory notifiable since 2001; and by the recent research into nosocomial norovirus outbreaks in Germany [22], [23].

*C. difficile* was the most frequently reported bacterial cause of HAI-outbreaks in our dataset, also reflected in a recent European-wide Point Prevalence Survey in acute care hospitals which identified *C. difficile* being among the most prevalent HAIs in Germany [3], [24]. Also Magill et al. recently found *C. difficile* to be the most common pathogen (causing 12.1% of HAIs) in a point prevalence survey of HAIs in U.S. hospitals [25].

Fifty percent of notified bacterial HAI-outbreaks were caused by MDROs. Selection bias may have led to an over-representation of MDRO-related outbreaks compared to those due to non-MDROs. Additionally, non-MDRO outbreaks are more likely to remain undetected. For example, a cluster of three *E. coli* urinary tract infections would usually not result in further investigation whereas three urinary tract infections with *E. coli* with an unusual resistance pattern would raise suspicion of an epidemiological link.

The median timeliness of forwarding notification data to RKI was within the legal time frame, although outliers were present. Since April 20, 2013 the IfSG stipulates that notifications of outbreaks have to be forwarded to RKI within two workdays, a requirement already fulfilled by most public health authorities.

Despite mandatory outbreak notification we assume that many outbreaks are not reported. This may be due to the narrow outbreak definition on the authority side but also to a lack of sensibility to the problem on the hospital side. In the previously mentioned survey among infection prevention and control staff to determine the frequency of outbreak investigations in US hospitals Rhinehart et al. found that 386 of 822 hospitals responding to the survey had performed outbreak investigations within the previous 24 months. [18] We received data on 485 outbreaks within 12 months from 2,045 German acute care hospitals [26]. A search within a public outbreak database (http://www.outbreak-database.com) [19] in February 2013 among 2,908 published outbreak reports revealed that since 2009 only three HAI-outbreaks in Germany have been published and so entered into this database [27]–[29]. To our knowledge only two additional outbreak reports from Germany not to be found in this outbreak database were published since 2009 [30], [31]. A plausible reason for the relatively low number of published outbreak reports can be partially illustrated by the prosecution and high public attention following publication of a detailed outbreak report from a neonatal and pediatric ICU in Germany [32], [33]. We hope that presentation of the HAI-outbreak surveillance data will encourage the responsible investigators to conduct outbreak investigations more regularly and to publish the results more frequently. On the other hand the low number of published investigations reveals the importance of the information collected by the surveillance system. Healthcare professionals may benefit from the surveillance results as they are informed about outbreaks in other facilities or regions.

Due to several limitations, the data on colonisations, symptomatic infections and fatalities should be interpreted with caution. It is likely that the number of colonised patients is underestimated as such data can only be obtained by active screening of exposed patients. Therefore, the true dimensions of an outbreak may not have been always captured by the notifications. Introduction or intensification of microbiological screening is recommended in suspected bacterial HAI-outbreaks [15], [16]; routine screening is advisable for certain patient risk groups including preterm infants with a birth weight <1500 g [34]. Nonetheless, differing screening modalities, and the time between infection of the index case and the initiation of screening activities may influence the number of detected colonised individuals. Furthermore, control measures such as cohort nursing and enhanced hand-hygiene may also influence the course of an outbreak and thereby influence our findings. Collection of information on these employed infection prevention and control measures in the surveillance system would enable systematic evaluation. However, the legal provisions for this national HAI-outbreak surveillance do not make this mandatory. A routine use of microbiological typing and molecular analysis of outbreak causing pathogens may provide added proof of actual outbreaks and disprove others.

Due to our definition of an HAI-outbreak, colonised cases are further underrepresented since outbreaks with fewer than two symptomatic infections did not have to be notified, and so did not meet our inclusion criteria. Therefore if there were 10 colonised cases with an epidemiological link including detection of the same outbreak strain, they would either not be reported or excluded from analysis.

The proportion of fatalities among cases may depend on the susceptibility and underlying comorbidities of patients, the setting of the outbreak, the detection of colonised individuals and the timeliness and effectiveness of implemented measures [35], [36].

Also, we identified that in a few notifications the sum of the number of colonised cases, symptomatic infected cases and fatalities was not equal to the total number of cases. This may be explained by following documentation errors: 1) the number of total cases was updated during follow-up notifications without updating numbers of colonised, symptomatic infected and fatalities; 2) double counting of infected cases that died as both an symptomatic infected case and a fatality.

The information collected on the causative pathogens was heterogeneous as it was documented in free text fields of the paper form. Nonetheless, we present a surveillance counting HAI-outbreaks within a country, whereas so far only estimates for HAI-outbreak incidence have been published. The integration of HAI-outbreak surveillance into the electronic surveillance system (SurvNet@RKI) has now been accomplished and will improve data-quality, as pre-defined categories replace almost all free text fields. As increasingly reliable data will be collected in the future, the potential of the surveillance system may be fully established, resulting in more accurate estimates of the distribution and of the role of single pathogens that are mainly responsible for HAI-outbreaks, helping to target control measures. Furthermore, reliable numbers on proportion of colonised and symptomatic infected patients and fatalities may one day enable clinicians and public health authorities to predict the potential severity of an outbreak at the time of its detection.

## Conclusion

Germany is among the first countries to implement a national surveillance system for HAI-outbreaks. Systematic nationwide data on HAI-outbreaks was not available in Germany prior to the implementation of this system in 2011. The system was overall well accepted. Within the first year one supra-regional outbreak was detected by the HAI-outbreak surveillance.

The results of the mandatory HAI-outbreak surveillance system describe the epidemiological and microbiological characteristics of common outbreaks, which may be beneficial for local outbreak management and control. In addition, pathogens with a high potential to cause large outbreaks or associated with high fatality rates may be identified in due time to establish effective prevention programmes.
